# Extracellular Vesicles and a Novel Form of Communication in the Brain

**DOI:** 10.3389/fnins.2016.00127

**Published:** 2016-03-31

**Authors:** Manuela Basso, Valentina Bonetto

**Affiliations:** ^1^Laboratory of Transcriptional Neurobiology, Centre for Integrative Biology, University of TrentoTrento, Italy; ^2^Department of Molecular Biochemistry and Pharmacology, IRCCS-Istituto di Ricerche Farmacologiche “Mario Negri,”Milano, Italy

**Keywords:** microvesicles, exosomes, neurodegeneration, cargo, nanotechnologies

## Abstract

In numerous neurodegenerative diseases, the interplay between neurons and glia modulates the outcome and progression of pathology. One particularly intriguing mode of interaction between neurons, astrocytes, microglia, and oligodendrocytes is characterized by the release of extracellular vesicles that transport proteins, lipids, and nucleotides from one cell to another. Notably, several proteins that cause disease, including the prion protein and mutant SOD1, have been detected in glia-derived extracellular vesicles and observed to fuse with neurons and trigger pathology *in vitro*. Here we review the structural and functional characterization of such extracellular vesicles in neuron-glia interactions. Furthermore, we discuss possible mechanisms of extracellular vesicle biogenesis and release from activated glia and microglia, and their effects on neurons. Given that exosomes, the smallest type of extracellular vesicles, have been reported to recognize specific cellular populations and act as carriers of very specialized cargo, a thorough analysis of these vesicles may aid in their engineering *in vitro* and targeted delivery *in vivo*, opening opportunities for therapeutics.

## Introduction

The central nervous system (CNS) is characterized by a reciprocal communication between its diverse cellular populations. Neurons, the effector cells, interact with astrocytes, microglia, oligodendrocytes and the vascular system to support their metabolic requests and respond to environmental stimuli. Neurons communicate with each other to transmit signals at synapses and release growth factors that influence the function and health of their targets. At the same time they interact with astrocytes that sense and respond to neuronal activity and participate to the re-uptake of neurotransmitters. Astrocytes also form end-foot processes, which constitute the blood-brain barrier, and regulate nutrients delivery based on neuronal activity. Through the capillaries, astrocytes sense external stimuli and can participate to the inflammatory response upon activation of microglia, the resident immune cells of the CNS. In the last two decades, numerous elegant papers established that the alteration of communication in the CNS is at the base of several neurodegenerative conditions. In Amyotrophic Lateral Sclerosis (ALS), the genetic removal of the mutant protein SOD1 in cell populations that are not usually vulnerable to the disease, such as astrocytes and microglia, was sufficient to delay the progression of the symptoms (Boillee et al., 2006; Yamanaka et al., 2008; Garden and La Spada, 2012). Accordingly, the expression of α synuclein or mutant huntingtin or the prion protein in astrocytes was sufficient to enhance neuronal vulnerability or induce disease (Ilieva et al., 2009). Finally, in Huntington's disease the sole expression of mutant huntingtin in cortical neurons, which are among the cells that dye during disease progression, is not sufficient to induce disease onset (Gu et al., 2005), confirming again that the disease can be induced by the interaction between different cell types.

**Figure 1:**
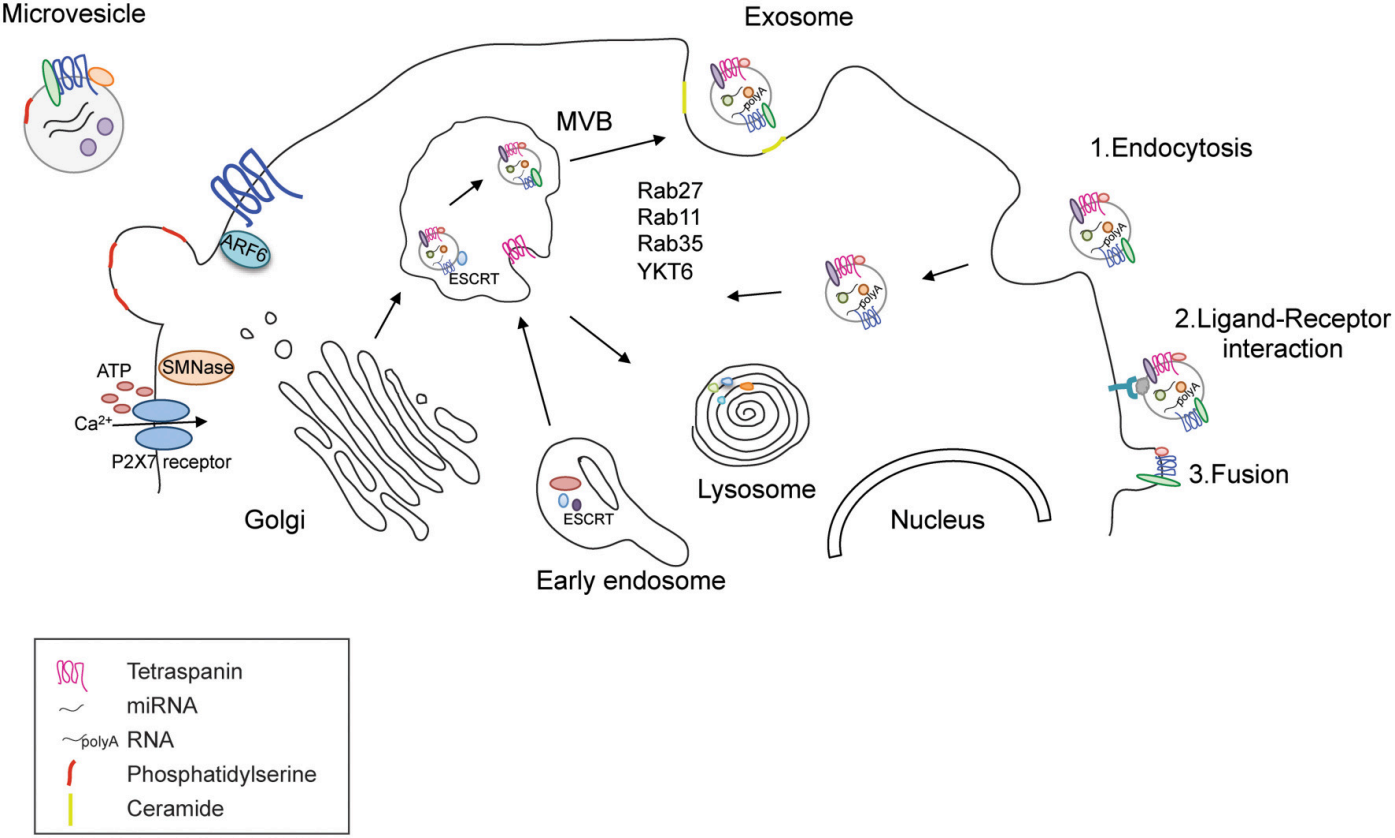
**Release and uptake of MVs and exosomes**. Functional EVs are released through two different pathways; MVs bud directly from the plasma membrane upon increase of extracellular ATP that opens the P2X7 receptor and allows the entrance of calcium. Their membrane is enriched in phosphatydil serine and tetraspanins. Their cargo contains and cytoplasmic proteins, RNAs and DNAs. Exosomes are derived from the late endosome or multivesicular bodies (MVB) where ESCRT coordinates the cargo loading and vesicle release. Rab GTPases, such as Rab27, 35, 11 and the SNARE protein YKY6 coordinate vesicle tethering and fusion to the plasma membrane. The cargo content comprises proteins and RNAs like in MVs. The presence of DNA is highly debated. Exosomes can enter in recipient cells trough endocytosis, ligand-receptor interaction (where both the ligand and the receptor are not clearly uncovered) or fusion with the plasma membrane.

One emerging mode of propagating such communication is through the release of extracellular vesicles (EVs). EVs can be divided into at least three types according to size and intracellular origin (Table 1). The first type of EV, termed exosomes, are small (50–200 nm) vesicles that are generated by the trafficking of multivesicular bodies (MVBs) from the cytosol to the cell surface; they are the most studied type of EV in terms of biogenesis and are characterized by RNA and protein cargo. In contrast, the second type of EV, termed microvesicles (MVs) or ectosomes (100–1000 nm), are derived from the plasma membrane and are released in response to specific stimuli, such as changes in ATP levels (Colombo et al., 2014; Figure 1). Finally, the third kind of EVs is called apoptotic bodies that are released during cell death (500–2000 nm). Their role in cell communication has not been explored so they will not be discussed further in this review.

**Table 1:** **Exosomes and MV main differences in biogenesis, properties, and functions**.

**Vesicle type**	**Properties**		**Biogenesis**	**Function**
	**Size**	**Lipids**		
Exosomes	50–200 nm	Ceramide	Late endosome (MVB)	Horizontal transmission of RNA and proteins
Microvesicles	50–1000 nm	Phosphatydilserine	Plasma membrane	Horizontal Transmission of DNA and RNA
Apoptotic bodies	500–2000 nm	Phosphatydilserine	Plasma membrane	Not yet reported

In the central nervous system (CNS), all cell types have been shown to release EVs that can either be taken up by neighboring cells or released into the cerebrospinal fluid (CSF) and blood (Colombo et al., 2012; Lugli et al., 2015). Although the function of EV uptake by neighboring cells remains uncovered, the detection of EVs in the CSF and plasma have raised the possibility that they may be useful for early diagnosis in neurodegenerative diseases (Colombo et al., 2012). Interestingly, pathogenic proteins such as prions, amyloid β peptide, superoxide dismutase, α-synuclein, and tau are released in association with EVs in a process that may spread pathogenic proteins throughout the body (Coleman and Hill, 2015). Genomic material has also been implicated in this cell-to-cell communication; reports of RNA contained in EVs in the CNS will be discussed along with EV biogenesis and release in neuronal cells, as well as EV function under both physiological and pathological conditions.

## Microvesicles and exosomes: different origin and different function?

Although differentiated by size and biogenesis pathway, MVs and exosomes share similar mechanisms of release and fusion with recipient cells, as has been recently reviewed (Cocucci and Meldolesi, 2015). MVs bud directly from the plasma membrane, which blebs and packages the cellular components in defined structures that are released into the extracellular environment upon cell activation (Turola et al., 2012). MVs contain distinct membrane regions of the cell of origin comprising receptors, proteins, and genomic material. Because the content can vary in different cell types or upon different stimuli, a major challenge has been related to the identification of common components of MVs that might enable their purification and characterization. One of the best markers is the presence of the lipid phosphatidylserine on the surface of MVs, which determines their ability to target recipient cells (Frey and Gaipl, 2011). The mechanisms of MV biogenesis and release are not completely understood in the CNS; several reports have implicated the endosomal sorting complex required for transport (ESCRT) pathway and ARF6 (Cocucci et al., 2009; Muralidharan-Chari et al., 2009; Gan and Gould, 2011) in this process, similar to exosomes. Interestingly, in cells expressing the purinergic receptor P2X7, such as microglia, an increase in the extracellular concentration of ATP induces the activation of the P2X7 receptor and the release of MVs and it is possible to observe an immediate pinching off of these vesicles from the plasma membrane (Bianco et al., 2005; Cocucci et al., 2009; Cocucci and Meldolesi, 2015). The transfer of MV cargo to recipient cells occurs via fusion or endocytosis of the vesicles into their target cell, but the mechanisms controlling the recognition of the target cell remain under investigation (Figure 1).

In contrast, exosomes derive from the inward budding of endosomal MVBs and are a more homogeneous population. The intraluminal vesicles contained in the MVBs can be either targeted to the lysosome or secreted as exosomes into the extracellular space. Accordingly, lysosome inhibition correlates with an increased release of α-synuclein from SH-SY5Y cells (Alvarez-Erviti et al., 2011a). The activation of either of these pathways is linked to the coordinated activity of the ESCRT complex with other proteins such as ALIX, TGS101, synthenin, and syndecan (Baietti et al., 2012). ESCRT is necessary to ubiquitinate proteins and direct them to the MVBs. It has been reported that silencing of early components of the ESCRT machinery decreases exosome production and alters their content (Colombo et al., 2013). Compelling evidence also supports the enzyme sphingomyelinase and the production of ceramide from raft-based microdomains rich in sphingolipids as important components of the vesicle budding process. Ceramide can self-associate through hydrogen bonding, thereby inducing the coalescence of microscopic rafts into a large membrane microdomain and facilitating exosome formation and release (Trajkovic et al., 2008). Other molecules that facilitate fusion of the MBV to the cell membrane are small GTPases such as RAB27A, RAB11, and RAB35 (Colombo et al., 2014). This is not surprising considering that Rab GTPase proteins are important regulators of intracellular trafficking in secretory pathways such as cargo selection in vesicle formation, vesicle transport, tethering, and docking (Binotti et al., 2016). It has been estimated that cells present specific sets of Rab proteins that control biogenesis, maturation, transition, and interaction of a subcellular organelle with other membranous compartments (Stenmark, 2009). Rab11 is the first small GTPase associated to exosome release (Savina et al., 2002). The overexpression of a dominant-negative mutant Rab11S25N inhibited exosome release suggesting a role of Rab11 in tethering and docking of MVBs to plasma membrane (Savina et al., 2005). At the same time, Rab11 has been recently observed in association with α-synuclein and its knockdown or the overexpression of the dominant negative form enhanced α-synuclein aggregation and toxicity in a cell model of Parkinson's disease (PD) (Chutna et al., 2014). To have a broader view of all the Rabs involved in exosome release, Ostrowski and colleagues used a small hairpin (sh)RNA screen to reveal that Rab27A and Rab27B where the two Rab GTPases controlling late endosome docking and fusion to the plasma membrane in HeLa cells (Ostrowski et al., 2010; Figure 1). Interestingly, Rab27b participates in synaptic vesicle exocytosis (Binotti et al., 2016) and in Drosophila it is part of a hub of Rabs that controls synaptic functions (Chan et al., 2011). Along this line, the inhibition of Rab35 leads to intracellular accumulation of endosomal vesicles and impairs exosome secretion in oligodendrocytes. Rab35 localizes to the surface of oligodendrocytes and plays a role in neurite outgrowth (Chevallier et al., 2009; Kobayashi and Fukuda, 2012, 2013; Kobayashi et al., 2014; Etoh and Fukuda, 2015), possibly mediated by its ability to control the release of exosomes as discussed in the paragraph “Functional role of EVs_Regulation of neuronal activity.” Other molecules that facilitate fusion of the MBV to the cell membrane are SNARE (SNAP Soluble NSF Attachment Protein Receptor) proteins like the YKT6 (Gross et al., 2012) even if SNAREs have been little investigated in the EV field (Colombo et al., 2014). Exosomes are usually released 10 min after the application of a specific stimulus, suggesting a delayed process compared to the budding of microvesicles (Soo et al., 2012; Cocucci and Meldolesi, 2015).

As is the case for MVs, the mechanisms determining specificity of exosome transmission to the target cells are not known, but the selectivity of neuronal exosomes to recognize other neuronal cells has been clearly shown (Chivet et al., 2013). Once exosomes are internalized into recipient cells, they can either merge into endosomes and form new exosomes or be degraded by the lysosome.

Both MVs and exosomes contain RNAs in the form of mRNA and non-coding RNA that is transmitted from one cell to the other. The important implication of horizontal transfer of vesicular mRNA and miRNA is a direct effect on epigenetic reprogramming of recipient cells or on the post-transcriptional control of specific genes. Of note, a recent paper reported that transmitted RNA is non-functional in recipient cells because it is degraded when released; instead plasmid DNA could be transmitted from one cell to another (Kanada et al., 2015). In line with this report, one recent study analyzed the differences between the cargos of MVs and exosomes, suggesting distinct functionalities (Keerthikumar et al., 2015b). Exosomes were mostly enriched with receptors and kinases that mediate signaling, whereas MVs presented centrosomal, ribosomal, and mitochondrial proteins, implicating a functional role in protein translation. An additional relevant difference concerns the involvement of exosomes in antigen presentation and in the transfer of both major histocompatibility complex molecules and antigens (Chang et al., 2013), suggesting a functional role for exosomes in immune regulation and in the interplay between the CNS and the immune system. In sum, exosomes and MVs can both deliver effector molecules such as proteins and RNAs to target cells, but their functional roles, which we are only beginning to appreciate, appear to be distinct.

Another aspect regards EV quantification that has proven challenging due to the heterogeneity in the size and composition of the population. The nanoparticle tracking analysis (NTA) is one of the most frequently utilized (Soo et al., 2012). NTA allows determining the mean size of the population of vesicles analyzed and the particle concentration estimation in the sample and can be paired to high-resolution flow cytometry (Nolte-'T Hoen et al., 2012; Maas et al., 2015). These techniques assess precisely the quality of the EV population, but not the quantity (Maas et al., 2015). Therefore, it is still not known if microvesicles and exosomes are differentially released in terms of total amount. Accordingly, we cannot infer how much protein is usually present per particle; for instance, the range is in the low micrograms per 10 ml of conditioned media derived from HEK293 cells (Jeppesen et al., 2014), 80–90 μg from 3 ml of plasma (Bonetto, unpublished results). Regarding the nucleic acid content, a detailed analysis has been performed in plasma of cancer patients (Chevillet et al., 2014). Surprisingly, the analysis revealed that there is on average only one copy of miRNA in each vesicle. The stoichiometric model proposes that either miRNAs may be distributed throughout the population of vesicles in a low occupancy/low miRNA content ratio or in a low occupancy/high miRNA concentration distribution. New experiments will be needed to assert the amount of cargo contained in EVs *in vivo* and their rate of release in physiological and pathological conditions.

## EV cargos in the CNS

Numerous targeted and omics studies have been carried out to identify the constituents of EVs in multiple organisms and cell types. Public online databases, such as ExoCarta (Mathivanan and Simpson, 2009; Keerthikumar et al., 2015a), EVpedia (Kim et al., 2015) and Vesiclepedia (Kalra et al., 2012), are available to provide EV molecular data mainly for proteins and nucleic acids, but also for lipids and metabolites. As of January 2015, the databases have reported 218 studies on exosomes and 123 studies on MVs (Vesiclepedia Version 3.1, release date January 2015).

### Proteins

Several proteomics studies have highlighted a set of proteins that are commonly found in exosome preparations (Table 2). These are (i) transmembrane or lipid-bound extracellular proteins, such as tetraspanins (e.g., CD9, CD63, CD81) and integrins (e.g., ITGB1); (ii) cytosolic proteins, such as endosomal or membrane binding (e.g., TSG101, ANXA5, ANXA2, FLOT1, RAC1), adapter (e.g., YWHAZ, YWHAE, SDCBP), and heat-shock (e.g., HSPA8, HSP90AA1) proteins; and (iii) extracellular proteins binding specifically or non-specifically to EV membranes (e.g., A2M, ALB). Intracellular proteins associated with compartments such as endoplasmic reticulum, nucleus, and mitochondria are absent or under-represented in exosomes, but may be present in other EVs (Lotvall et al., 2014; Keerthikumar et al., 2015b). EV loading is thought to be an active process controlled through a variety of pathways, ESCRT-dependent and independent, most of which are still not fully understood (Villarroya-Beltri et al., 2013a, 2014). In fact, EVs are enriched in specific proteins, lipids and RNAs. However, the most commonly found proteins in EVs (Table 2), excluding the ones associated with the membrane/vesicle trafficking, are also the most abundant proteins of the cell (highest protein copies per cell; Beck et al., 2011), arguing that a sorting process is applied to all cargos. The relative proportions of the different proteins appear to vary depending on conditions and types of EVs (Keerthikumar et al., 2015b). One recent proteomic study compared exosomes and MVs from neuroblastoma cell lines and identified candidate protein markers that might aid in discriminating between them: VPS24, VPS32, VPS36, CD81, TSPAN9, TSPAN14, ANXA7, synthenin, and ITGA3 in exosomes and RACGAP1, PDIA3, SPTBN2, MUC19, UBR4, KRT18, KIF14, KIF4A, VIM, RPS9, RPS18, and MMP2 in MVs (Keerthikumar et al., 2015b). However, since exosomes and MVs are physically very similar, results with purified vesicles using current technologies have yet to be considered with caution. Notably, most studies use EVs generated by endothelial cells, stem cells, or tumor cells because neuronal cells generally release low amounts of EVs, and are difficult to detect based on current technologies. Unique proteins that reflect the specialized function of the cells of origin have been identified; for example, myelin proteins have been found in EVs from oligodendrocytes (Kramer-Albers et al., 2007). Other studies have examined EVs in the context of disease, identifying microglia-derived EVs in the CSF of patients with multiple sclerosis using isolectin B4 labeling (Verderio et al., 2012). Neurofilament proteins (namely NEFL and NEFM) have been found in EVs isolated from human CSF (Chiasserini et al., 2014). However, true cell-specific markers have not yet been identified, despite their clear utility in understanding the contribution and impact of the different types of EVs in the CNS.

**Table 2:** **List of the top 50 most commonly identified proteins in exosome preparations**.

	**Protein name**	**Gene**	**Times^a^**	**Function**
**1**	**CD9 antigen**	***CD9***	**98**	Integral membrane protein
**2**	Heat shock cognate 71 kDa protein^*^	***HSPA8***	**96**	Protein folding
3	Programmed cell death 6 interacting protein	*PDCD6IP*	96	Adapter in vesicle trafficking
4	Glyceraldehyde-3-phosphate dehydrogenase^*^	*GAPDH*	95	Glycolysis
5	Actin, beta^*^	*ACTB*	93	Cytoskeletal
**6**	Annexin A2^*^	***ANXA2***	**83**	Vesicle trafficking
**7**	**CD63 antigen**	***CD63***	**82**	Integral membrane protein
**8**	**Syntenin-1**	***SDCBP***	**78**	Protein membrane scaffolding
9	Alpha-enolase^*^	*ENO1*	78	Glycolysis
10	Heat shock protein HSP 90-alpha^*^	*HSP90AA1*	77	Protein folding
**11**	**Tumor susceptibility gene 101 protein**	***TSG101***	**75**	Vesicle trafficking
12	Pyruvate kinase PKM^*^	*PKM*	72	Glycolysis
13	L-lactate dehydrogenase A chain^*^	*LDHA*	72	Glycolysis/ Krebs cycle
14	Elongation factor 1-alpha 1^*^	*EEF1A1*	71	Translation
15	14-3-3 protein zeta/delta^*^	*YWHAZ*	69	Adapter in signaling
16	Phosphoglycerate kinase 1^*^	*PGK1*	69	Glycolysis
17	Eukaryotic elongation factor 2 kinase^*^	*EEF2*	69	Translation
18	Fructose-bisphosphate aldolase A^*^	*ALDOA*	69	Glycolysis
19	Heat shock protein 90kDa alpha class B member 1^*^	*HSP90AB1*	67	Protein folding
**20**	Annexin A5^*^	***ANXA5***	**67**	Vesicle trafficking
21	Fatty acid synthase	*FASN*	66	Fatty acid biosynthesis
22	14-3-3 protein epsilon^*^	*YWHAE*	65	Adapter in signaling
23	Clathrin heavy chain 1	*CLTC*	64	Component of coated vesicles
**24**	**CD81 antigen**	***CD81***	**64**	Integral membrane protein
25	Serum albumin	*ALB*	63	Extracellular (aspecific binding?)
26	Valosin-containing protein^*^	*VCP*	62	Protein degradation
27	Triosephosphate isomerase^*^	*TPI1*	62	Glycolysis
**28**	Peptidylprolyl isomerase A (cyclophilin A)^*^	***PPIA***	**62**	Protein folding
29	Moesin^*^	*MSN*	62	Cytoskeletal
30	Cofilin-1^*^	*CFL1*	62	Cytoskeletal
31	Peroxiredoxin-1^*^	*PRDX1*	61	Redox regulation
32	Profilin-1^*^	*PFN1*	61	Cytoskeletal
33	Ras-related protein Rap-1b	*RAP1B*	60	Small GTPase
**34**	**Integrin, beta-1**	***ITGB1***	**60**	Integral membrane protein
35	78 kDa glucose-regulated protein	*HSPA5*	58	Protein folding
36	4F2 cell-surface antigen heavy chain	*SLC3A2*	57	Integral membrane protein
37	Histone H4^*^	*HIST1H4A*	57	DNA binding
38	Guanine nucleotide-binding protein, beta 2^*^	*GNB2*	57	Small GTPase
39	Sodium/potassium-transporting ATPase alpha-1	*ATP1A1*	57	Metabolic process
40	14-3-3 protein theta^*^	*YWHAQ*	56	Adapter in signaling
**41**	**Flotillin-1**	***FLOT1***	**56**	Vesicle trafficking
42	Filamin-A	*FLNA*	56	Cytoskeletal
43	Chloride intracellular channel protein 1^*^	*CLIC1*	56	Ion channel
44	T-complex protein 1 subunit beta^*^	*CCT2*	56	Protein folding
45	CDC42 small effector protein 1	*CDC42*	55	Small GTPase
46	14-3-3 protein gamma	*YWHAG*	54	Adapter in signaling
47	Alpha-2-macroglobulin	*A2M*	54	Extracellular (aspecific binding?)
48	Tubulin alpha-1B chain^*^	*TUBA1B*	53	Cytoskeletal
49	Ras-related C3 botulinum toxin substrate 1	*RAC1*	53	Small GTPase
50	Galectin-3-binding protein	*LGALS3BP*	53	Cell adhesion

a *Number of times identified in the studies collected by the Exocarta database (Mathivanan and Simpson, 2009; Keerthikumar et al., 2015a), release date 29 July 2015; proteins indicated in bold are often used as exosome markers*.

* *Very abundant proteins in human cells [protein copies per cell > 100 × 10^6^ (Beck et al., 2011)]*.

Most studies of EVs in the CNS have examined EVs isolated from cultured cell media (e.g., neurons, astrocytes, microglia, and oligodendrocytes); very few have used CSF samples. Among these studies, Vella et al. first identified the prion protein in EVs from ovine CSF (Vella et al., 2008a). Later proteomic analyses of EVs isolated from human CSF provided limited information (Harrington et al., 2009; Street et al., 2012). More recently, Chiasserini et al. applied high resolution MS/MS-based proteomics to the analysis of EVs isolated from pooled samples of CSF and built a dataset of proteins that provide a basis for biomarker studies in neurological diseases (Chiasserini et al., 2014). Interestingly, EVs contain several proteins that have relevance to CNS disease, including all major neuropathological hallmarks of neurodegenerative diseases, such as amyloid β peptide and tau/phosphorylated tau for Alzheimer's disease (AD), α-synuclein for PD, and SOD1/mutant SOD1 and TDP-43 for amyotrophic lateral sclerosis (ALS) (Table 3). The reason for this it has not yet been understood. We can hypothesize that cells use EV secretion as an alternative pathway to remove aggregate-prone pathological proteins when the protein clearing system is dysfunctional, which is a common feature of neurodegenerative diseases. For example we reported increased exosome secretion in astrocytes expressing mutant SOD1 (Basso et al., 2013), which is known to escape the cell degradation machinery and impair the proteasomal system and autophagy in experimental models of ALS (Bendotti et al., 2012; Chen S. et al., 2012). Moreover, secretion of toxic α-synuclein oligomers via exosomes is strongly influenced by autophagic activity, with enhanced release with an autophagic inhibitor and decrease release with an autophagic enhancer (Danzer et al., 2012). It is also possible that aggregation or higher-order oligomerization act as a general sorting signal to target certain proteins into EVs providing an effective means for clearing damaged and potentially toxic proteins from the cells (Vidal et al., 1997; Fang et al., 2007).

**Table 3:** **A selection of proteins identified in EVs isolated from cultured cell media (neurons, astrocytes, microglia, oligodendrocytes) and CSF that are relevant for the CNS under physiological and pathological conditions**.

**Gene**	**Protein name**	**N^a^**	**A^b^**	**M^c^**	**O^d^**	**CSF**	**References^e^**
*ANG*	Angiogenin					X	Chiasserini et al., 2014
*APOE*	Apolipoprotein E	X				X	Harrington et al., 2009; Marimpietri et al., 2013
*APP*	Amyloid beta A4 protein	X		X		X	Rajendran et al., 2006; Harrington et al., 2009; Joshi et al., 2014
*CD13*	Aminopeptidase N			X			Potolicchio et al., 2005
*CP*	Ceruloplasmin	X				X	Faure et al., 2006; Chiasserini et al., 2014
*CNP*	2′,3′-cyclic-nucleotide 3'-phosphodiesterase	X			X	X	Kramer-Albers et al., 2007; Marimpietri et al., 2013; Chiasserini et al., 2014
*EAAT1*	Excitatory amino acid transporter 1	X					Faure et al., 2006
*GRIA2*	Glutamate receptor 2	X					Faure et al., 2006
*GRIA3*	Glutamate receptor 3	X					Faure et al., 2006
*GRIA4*	Glutamate receptor 4					X	Chiasserini et al., 2014
*HNRNPA1*	Heterogeneous nuclear ribonucleoprotein A1					X	Chiasserini et al., 2014; Keerthikumar et al., 2015a
*IL1B*	Interleukin-1 beta			X			Bianco et al., 2005
*MBP*	Myelin basic protein				X	X	Kramer-Albers et al., 2007; Chiasserini et al., 2014
*MCT1*	Monocarboxylate transporter 1	X		X			Potolicchio et al., 2005; Marimpietri et al., 2013
*MMP2*	Matrix metallopeptidase-2		X			X	Sbai et al., 2010; Chiasserini et al., 2014
*MMP9*	Matrix metallopeptidase-9		X				Sbai et al., 2010
*MOG*	Myelin oligodendrocyte glycoprotein				X	X	Kramer-Albers et al., 2007; Chiasserini et al., 2014
*NEFL*	Neurofilament light polypeptide					X	Chiasserini et al., 2014
*NEFM*	Neurofilament medium polypeptide					X	Harrington et al., 2009
*PAR4*	Prostate apoptosis response 4 protein (PAR-4)		X				Wang et al., 2012
*PARK7*	Protein deglycase DJ-1					X	Chiasserini et al., 2014
*PFN1*	Profilin-1					X	Chiasserini et al., 2014
*PLP1*	Myelin proteolipid protein				X	X	Kramer-Albers et al., 2007; Chiasserini et al., 2014
*PPIA*	Peptidyl-prolyl cis-trans isomerase A (Cyclophilin A)	X	X^f^	X	X	X	Potolicchio et al., 2005; Faure et al., 2006; Kramer-Albers et al., 2007; Chiasserini et al., 2014
*PRNP*	Prion protein	X				X	Faure et al., 2006; Vella et al., 2008a
*SOD1*	Superoxide dismutase [Cu-Zn]	X^g^	X^g^			X	Gomes et al., 2007; Basso et al., 2013; Chiasserini et al., 2014
*SNCA*	Alpha-synuclein	X					Emmanouilidou et al., 2010
*SYN1*	Synapsin-1		X			X	Wang et al., 2011; Chiasserini et al., 2014
*MAPT*	Microtubule-associated protein tau	X^h^		X		X^i^	Saman et al., 2012; Asai et al., 2015
*TARDBP*	TAR DNA-binding protein 43 (TDP-43)	X					Nonaka et al., 2013
*TUBA4A*	Tubulin alpha-4A chain	X				X	Chiasserini et al., 2014; Keerthikumar et al., 2015a
*VCP*	Valosin-containing protein	X	X			X	Basso et al., 2013; Chiasserini et al., 2014; Keerthikumar et al., 2015a
*VEGFA*	Vascular endothelial growth factor A	X	X				Schiera et al., 2007; Proia et al., 2008

a *N, neuronal cells (primary cultures or cell lines)*.

b *A, astrocytes*.

c *M, microglia*.

d *O, oligodendrocytes*.

e *References, the first published evidence*.

f *Unpublished evidence*.

g *Detected wild-type and mutant SOD1*.

h *Detected tau and phosphorylated tau*.

i *Detected only phosphorylated tau*.

The findings that misfolded/aggregated proteins can be secreted through EVs have made these vesicles extremely attractive as a source of biomarkers for neurodegenerative diseases, with relevant publications recently emerging from studies of peripheral biofluids (Shi et al., 2014; Fiandaca et al., 2015; Goetzl et al., 2015; Tomlinson et al., 2015). Although the fraction of CNS-specific EVs is likely limited in peripheral biofluids, peripheral EV protein biomarkers could be useful to monitor systemic alterations that reflect CNS alterations and at the same time impact CNS function, as was previously demonstrated with peripheral blood mononuclear cell protein biomarkers in ALS (Nardo et al., 2011). Interestingly, several proteins, including VCP and PPIA, which have been associated with ALS and frontotemporal lobar degeneration (Johnson et al., 2010; Lauranzano et al., 2015) are among the most commonly identified proteins in EVs (Table 3). These proteins are present in both exosomes and MVs from neurons, glial cells, and human CSF; however, no reports have demonstrated the functional implications of these common proteins being released in EVs. Other EV proteins causally linked to neurodegenerative diseases include APOE (AD), ANG, PFN1, HNRNPA1, and TUBA4A (ALS), and PARK7 (PD; Table 3). EVs can also contain proteins that play roles in neuroprotection (e.g., VEGF, SYN, and CP), neuronal function (e.g., EAAT1, GRIA2-4), neuroinflammatory response (e.g., MMP2, MMP9 and IL1B), and apoptosis (e.g., PAR4; Table 3).

### Nucleic acids

The first report of RNA in EVs (Valadi et al., 2007) opened a new perspective in the field, suggesting that not only proteins but also genomic material (namely mRNA and miRNA) could be exchanged between cells. MiRNAs are a class of small RNA molecules that regulate gene expression by binding to mRNAs and triggering their degradation or inhibiting their translation. Considering that miRNAs can control the expression of multiple mRNAs, it is believed that the horizontal transmission of miRNAs through EVs play a functional role in intercellular communication (Chen X. et al., 2012; Ramachandran and Palanisamy, 2012). An additional interest was the possibility of using EV-derived small non-coding RNAs as novel biomarkers for neurodegeneration (Rao et al., 2013), as EVs can be recovered not only from CSF but also from peripheral sources such as blood and urine. Further studies, reviewed in (Rao et al., 2013), showed that plasma exosomes contained RNA derived from neuronal cells, potentially mirroring the activities of the CNS and offering the opportunity for non-invasive analysis. At the same time, these studies shed light on several technical challenges related to the observation that different extraction techniques of similar samples could lead to diverse miRNA detection. To minimize the incongruence between different laboratories, the International Society of Extracellular Vesicles published guidelines and gold standard protocols that should be followed when working with EVs (Lotvall et al., 2014). To date, a plethora of small non-coding RNAs have been observed in EVs, including vault RNA, Y-RNA, piwi RNA, small nucleolar RNA, small nuclear RNA, and tRNA, although there appears to be preferential sorting for selected miRNAs (Janas et al., 2015). This sorting may be controlled by the affinity between miRNAs and specific RNA-binding proteins that deliver them to the raft-like region of the MVB limiting membrane. In particular, the protein heterogeneous nuclear riboprotein A2B1 (hnRNP2AB1) recognizes and binds specific RNA motifs in miRNAs (called EXOmotifs, Table 4) and controls their sorting into exosomes when sumoylated (Villarroya-Beltri et al., 2013b). Interestingly, hnRNPA2B1 is involved in RNA trafficking in neurons (Carson and Barbarese, 2005), and mutations in hnRNPA2B1 cause a multisystem proteinopathy called inclusion body myopathy with Paget disease and frontotemporal dementia (Kim et al., 2013). Furthermore, hnRNPA2B1 interacts with another RNA-binding protein, TDP-43, whose presence in protein inclusions is a hallmark of ALS and frontotemporal dementia, and with PPIA, a biomarker of ALS and a key regulator of TDP-43 function (Nardo et al., 2011; Lauranzano et al., 2015). Another observation confirming that specific miRNAs are loaded in EVs comes from the comparative analysis of miRNAs contained in EVs derived from HEK293T cells, mesenchymal stem cells, macrophages, and immune cells, showing that miR-451 is one of the most commonly released miRNAs across all different sources (Guduric-Fuchs et al., 2012). MiR-451 controls nine genes, including *MYC* and *AKT*, and is increased in neuronal tissues of mice that have undergone traumatic brain injury (Meissner et al., 2015) and downregulated in neuronal cells treated with the antioxidant EPO (Alural et al., 2014), suggesting that its secretion via EVs could be important in acute damage. Other properties that determine the selective loading of miRNA cargo are related to the presence of a lipid-bilayer binding motif within the RNA sequence and RNA hydrophobic modifications that facilitate RNA binding to the EV membrane (Janas and Yarus, 2006).

**Table 4:** **EXOMotifs. Specific RNA motifs recognized by hnRNPA2B1 for cargo loading in EVs**.

	**RNA Motif**
1	GGAG
2	UGAG
3	UGCG
4	UGGG
5	UGAC
6	UGCC
7	UGGC
8	GGCG
9	GGGG
10	GGAC
11	GGGG
12	GGCC
13	GGGC
14	CCCU
15	CCCG
16	CCCA
17	UCCU
18	UCCG
19	UCCA
20	GCCU
21	GCCG
22	GCCA

The miRNAs released in EVs are functional and activate specific signals in target cells, as in the case of miR-124a, which is transferred from neurons to astrocytes and regulates the levels of the excitatory amino acid transporter 2, important in synaptic modulation (Morel et al., 2013). Another interesting example is the release of EVs rich in miRNAs during synaptic depolarization that leads to a local decrease of miRNAs at the synaptic cleft and a global reduction in postsynaptic gene silencing to facilitate a rapid increase in local translation (Goldie et al., 2014).

To date, few studies have been performed in neurons to identify exosomal non-coding RNAs, and reports are still lacking regarding the potential identification of biomarkers for neurodegenerative diseases. One pilot study of EV-derived miRNAs purified from cells infected with the prion protein reported an increase in the release of several miRNAs such as let-7b, miR-146a, miR-103, miR-125a-5p, miR-342-3p also shown to be dysregulated in human samples obtained from patients affected by the prion disease (Bellingham et al., 2012). Of note, it has been recently estimated that only a small percentage of exosomes contain miRNAs, supporting the hypothesis that only specific exosomes carry a full load of miRNAs and transport them to selected targets (Chevillet et al., 2014).

## Functional roles of EVs

The identification of EV cargos is essential to understand the biological relevance of this system in the CNS, but such studies must be paired with investigations into functional roles for EVs. In this regard, numerous observations have been reported in several aspects of neuronal biology ranging from development to degeneration. Here we discuss the most recent observations regarding the role of EVs in synaptic activity and in physiological and pathological conditions, with a final discussion of the most recent technologies developed to address this important question.

### Regulation of neuronal activity

The first evidence of a functional role for EVs in the CNS was obtained *in vitro*, in which EVs were shown to contribute to the modulation of the synaptic activity. Initial studies reported that the release of EVs increased when neurons were depolarized with KCl (Faure et al., 2006) or treated with bicullin, an agonist of GABA_*A*_ receptors (Lachenal et al., 2011). Neuronal exosomes carry glutamate receptor (GluR2) subunits and are thought to participate in synaptic plasticity, regulating the amount of postsynaptic AMPA and NMDA receptors. Notably, a subsequent paper from the same group reported that exosomes released by neurons were able to recognize their target cells, suggesting that these released vesicles likely play a specific function (Chivet et al., 2013). For instance, exosomal synaptotagmin 4 is released by presynaptic terminals and transmitted to postsynaptic cells, activating retrograde signaling and synaptic growth (Korkut et al., 2013). At the same time, exosomes derived from oligodendrocytes can also be internalized by neurons at axonal and somatodendritic sites and can protect neurons from insults (Fruhbeis et al., 2013) by inducing the transcription of genes involved in the neuronal antioxidant response, such as catalase and superoxide dismutase (Fröhlich et al., 2014). Interestingly, neuronal activity appears to be necessary to induce the exchange of EVs, given the observation that calcium chelation at the synaptic cleft or inhibition of NMDA receptors is sufficient to fully abrogate exosome release (Fruhbeis et al., 2013). Microglia can also participate in the modulation of synaptic activity via MVs (Antonucci et al., 2012). MVs that are released from microglia interact with the neuronal plasma membrane and enhance spontaneous and evoked excitatory transmission by inducing sphingolipid metabolism in neurons (Antonucci et al., 2012); the increase in ceramide upregulates glutamate secretion in culture and initiates synaptic activity. Similarly, transport of endocannabinoids via MVs stimulates type 1 cannabinoid receptors and inhibits presynaptic transmission in GABAergic neurons (Gabrielli et al., 2015).

Astrocytes are also involved in the release of EVs and in modulation of synaptic activity. Astrocytes scavenge extracellular glutamate through membrane excitatory amino acid transporters (EAAT-1 and EAAT-2) in a process that is essential for neurotransmission. Accordingly, loss of glutamate homeostasis has been linked to neurodegeneration (Kim et al., 2011). The astrocyte glutamate transporters EAAT-1 and EAAT-2 have been identified in EVs upon activation of protein kinase C, which normally controls their subcellular localization (Gosselin et al., 2013); however, a direct role in synapse modulation remains to be established. Neurons also control their own synaptic activity and dendrite growth through the release of EVs that contain specific miRNAs that locally regulate protein translation (Goldie et al., 2014) or induce the expression of the EAAT-2 in astrocytes; specifically, miR-124 is released by neurons and can modulate the expression of EAAT-2 in neighboring astrocytes (Morel et al., 2013).

### Involvement in neurodegenerative processes: EV and the prion-like hypothesis of neurodegenerative diseases

The prion protein was the first protein to be detected in exosomes of ovine CSF infected by prion disease (Vella et al., 2008a), followed by phosphorylated tau (Saman et al., 2012) and amyloid β in models of AD (Yuyama et al., 2015) and α-synuclein in patients with PD (Shi et al., 2014; Yang et al., 2015). Notably, all of these proteins have shown a propensity to assume a prion-like behavior of changing the conformation of other proteins in tissue and propagate disease (Ghidoni et al., 2008; Coleman and Hill, 2015). Accordingly, the injection of exosomes containing prions induced prion propagation in wild-type recipient cells and induced prion disease when inoculated into mice (Fevrier et al., 2004; Vella et al., 2007, 2008b). Similar EV toxicity was reported for α-synuclein in PD (Emmanouilidou et al., 2010) and for mutant SOD1 (Basso et al., 2013; Grad et al., 2014) and TDP-43 (Ding et al., 2015) in ALS. Nevertheless, it is important to consider that definitive studies regarding the necessity and sufficiency of the mutant proteins in EVs for the onset of neurodegeneration have not been reported; indeed, other cargos, such as metabolites, proteins, or RNAs could play a primary role in the propagation of pathology. Consistent with this concept, the reduction of exosome release by inhibition of neutral sphingomyelinase 2 by GW4869 in an *in vivo* model of AD lowered the amount of amyloid β plaques observed in brain sections (Dinkins et al., 2014). The same treatment also proved to be effective in another *in vivo* AD model, characterized by the spreading of aggregated tau from the entorhinal cortex to the hippocampal region; in this study, the authors demonstrated that aggregated tau was phagocytosed by microglia and released in EVs. The depletion of microglia or inhibition of EV release was sufficient to halt the spreading of the disease (Asai et al., 2015). Similarly, exosomes purified from astrocytes expressing mutant SOD1 were sufficient to induce motor neuron death (Basso et al., 2013). This may indicate that glial cells that in normal conditions are secreting trophic and protective factors for neurons, under pathological conditions are changing their phenotype and contribute to spreading and toxicity trough EV secretion. It would be interesting to test whether this happens also with oligodendrocytes and in different models of neurodegenerative diseases. Indeed EVs purified from other sources have also showed protective effects. For example, exosomes derived from N2a cells and injected intracerebroventricularly in the APP^SweInd^ model of AD reduced amyloid β levels, amyloid deposition, and amyloid β-mediated synaptotoxicity in the hippocampus. This beneficial effect correlated with an increase in glycosphingolipids in exosomes, essential to scavenge amyloid β extracellularly and facilitate its clearance (Yuyama et al., 2014). An additional study reported that exosomes purified from the plasma of PD patients were internalized into primary neuronal cultures and could protect neurons exposed to toxic stimuli (Tomlinson et al., 2015). Considering these data, caution must be used in interpreting the protective or pathogenic effects of EVs in neurodegeneration.

### Establishment of new tools to test EV functionality *In vivo*

The most significant promise of EVs is their potential therapeutic application. EVs are stable in the blood and can therefore be injected into the body, cross the blood-brain barrier, and reach their targets. Ideally, it might be possible to induce the production of massive amounts of EVs from specific cells, purify them, and introduce particular molecules such as siRNAs (El-Andaloussi et al., 2012), anti-miRs, or lipids (Yuyama et al., 2014) to restore the lipid dyshomeostasis that is typical of neurodegenerative conditions. Elegant strategies have been developed to monitor EVs *in vivo*. One recent paper (Wiklander et al., 2015) characterized the biodistribution of EVs in mice after systemic delivery. In this study, EVs were isolated from three different mouse cell sources and labeled with a lipophilic dye, demonstrating that the origin of the cells and the route of administration, along the dose of injected EVs, influenced the biodistribution pattern. For example, EVs derived from dendritic cells preferentially accumulated in the spleen, and the best route of delivery for the spleen was intravenous injection. Notably, the specific delivery of EVs to the CNS required the presence of an RVG peptide, derived from the acetylcholine receptor, on the EV membrane. The use of these engineered EVs led to the effective silencing of *BACE1*, an approach that has therapeutic promise in AD (Alvarez-Erviti et al., 2011b), or silencing of α-synuclein, which reduced the formation of intracellular inclusions in an *in vivo* model of PD (Cooper et al., 2014).

An additional compelling tool to allow *in vivo* tracking is the labeling of EVs with Gaussia luciferase for noninvasive bioluminescence imaging (Lai et al., 2014b). In one recent study, EVs were engineered to display a membrane reporter, termed EV-GlucB, consisting of Gaussia luciferase fused with a biotin acceptor domain, which is biotinylated *in vivo* by biotin ligases. The luciferase is revealed after administration of the substrate and monitored with *in vivo* bioluminescence imaging technology. At the same time, biotin on the EV surface can be conjugated to labeled streptavidin and imaged with several techniques such as fluorescence-mediated tomography (Lai et al., 2014a). Such new technologies will be particularly useful as researchers move into more translational areas of EV research.

## Future perspectives

The field of EVs is continuously growing and has generated significant excitement, as testified by the recent exponential increase in the number of EV-related publications. The involvement of EVs in the modulation of synaptic activity, as well as their functional roles in the spreading of neurodegenerative diseases, highlight the importance of studying and understanding how to engineer EVs to counteract their toxic effects. Additional studies are necessary to uncover the mechanisms leading to EV formation in the CNS. Although ceramide and sphingomyelinase 2 are certainly playing an important role in EV formation, it is unlikely that they can orchestrate the choice of cargo. ESCRT also appears to be important for exosome formation; notably, ESCRT dysfunction along with aberrant endosomal trafficking and improper ubiquitination and deubiquitination are related to neurodegeneration. STAM1, HRS, and AMSH, three proteins of the ESCRT complex, induce neuronal loss in the hippocampus when knocked out in mice. Knockout mice for STAM1/STAM2 and UBPY, other ESCRT proteins, are embryonic lethal (Kapuralin et al., 2015).

Another open question relates to the specificity of EV cargos in the CNS. In this regard, only a thorough characterization of their contents, including the metabolites and lipids that are not typically analyzed, will help to define EV functions. Other unresolved questions relate to the characterization of the proteins localized on the extracellular membrane of EVs that serve as receptors and mediate the recognition of the correct target cell.

EVs have also the potential to identify specific biomarkers in the CNS to monitor the outcome of neurodegenerative conditions. Given their relatively stable biophysical properties, EVs released by the CNS, muscles, or other cells affected by disease can readily circulate through the body and be found in biological fluids, including blood (Verderio et al., 2012). As the generation and release of EVs can be an extremely rapid cellular process, reflecting dynamic cellular states and eventually specific disease stages, EVs have great potential to report on disease progression with high sensitivity and specificity. In particular, plasma concentration of exosomes and their biochemical properties may be useful parameters, although these must be verified in clinically well-characterized longitudinal cohorts of patients and controls with the aim of identifying a reliable fingerprint of disease progression. Biomarkers for disease progression could shorten the duration of clinical trials, reducing the cost and time necessary to bring important therapies to patients.

## Author contributions

All authors listed, have made substantial, direct and intellectual contribution to the work, and approved it for publication.

### Conflict of interest statement

The authors declare that the research was conducted in the absence of any commercial or financial relationships that could be construed as a potential conflict of interest.

## References

[B1] Alural B. Duran G. A. Tufekci K. U. Allmer J. Onkal Z. Tunali D.. (2014). EPO mediates neurotrophic, neuroprotective, anti-oxidant, and anti-apoptotic effects via downregulation of miR-451 and miR-885-5p in SH-SY5Y neuron-Like cells. Front. Immunol. 5:475. 10.3389/fimmu.2014.00475 25324845 PMC4179732

[B2] Alvarez-Erviti L. Seow Y. Schapira A. H. Gardiner C. Sargent I. L. Wood M. J.. (2011a). Lysosomal dysfunction increases exosome-mediated alpha-synuclein release and transmission. Neurobiol. Dis. 42, 360–367. 10.1016/j.nbd.2011.01.029 21303699 PMC3107939

[B3] Alvarez-Erviti L. Seow Y. Yin H. Betts C. Lakhal S. Wood M. J. (2011b). Delivery of siRNA to the mouse brain by systemic injection of targeted exosomes. Nat. Biotechnol. 29, 341–345. 10.1038/nbt.1807 21423189

[B4] Antonucci F. Turola E. Riganti L. Caleo M. Gabrielli M. Perrotta C.. (2012). Microvesicles released from microglia stimulate synaptic activity via enhanced sphingolipid metabolism. EMBO J. 31, 1231–1240. 10.1038/emboj.2011.489 22246184 PMC3297996

[B5] Asai H. Ikezu S. Tsunoda S. Medalla M. Luebke J. Haydar T.. (2015). Depletion of microglia and inhibition of exosome synthesis halt tau propagation. Nat. Neurosci. 18, 1584–1593. 10.1038/nn.4132 26436904 PMC4694577

[B6] Baietti M. F. Zhang Z. Mortier E. Melchior A. Degeest G. Geeraerts A.. (2012). Syndecan-syntenin-ALIX regulates the biogenesis of exosomes. Nat. Cell Biol. 14, 677–685. 10.1038/ncb2502 22660413

[B7] Basso M. Pozzi S. Tortarolo M. Fiordaliso F. Bisighini C. Pasetto L.. (2013). Mutant copper-zinc superoxide dismutase (SOD1) induces protein secretion pathway alterations and exosome release in astrocytes: implications for disease spreading and motor neuron pathology in amyotrophic lateral sclerosis. J. Biol. Chem. 288, 15699–15711. 10.1074/jbc.M112.425066 23592792 PMC3668729

[B8] Beck M. Schmidt A. Malmstroem J. Claassen M. Ori A. Szymborska A.. (2011). The quantitative proteome of a human cell line. Mol. Syst. Biol. 7, 549. 10.1038/msb.2011.82 22068332 PMC3261713

[B9] Bellingham S. A. Coleman B. M. Hill A. F. (2012). Small RNA deep sequencing reveals a distinct miRNA signature released in exosomes from prion-infected neuronal cells. Nucleic Acids Res. 40, 10937–10949. 10.1093/nar/gks832 22965126 PMC3505968

[B10] Bendotti C. Marino M. Cheroni C. Fontana E. Crippa V. Poletti A.. (2012). Dysfunction of constitutive and inducible ubiquitin-proteasome system in amyotrophic lateral sclerosis: implication for protein aggregation and immune response. Prog. Neurobiol. 97, 101–126. 10.1016/j.pneurobio.2011.10.001 22033150

[B11] Bianco F. Pravettoni E. Colombo A. Schenk U. Moller T. Matteoli M.. (2005). Astrocyte-derived ATP induces vesicle shedding and IL-1 beta release from microglia. J. Immunol. 174, 7268–7277. 10.4049/jimmunol.174.11.7268 15905573

[B12] Binotti B. Jahn R. Chua J. J. (2016). Functions of rab proteins at presynaptic sites. Cells 5:E7. 10.3390/cells5010007 26861397 PMC4810092

[B13] Boillee S. Yamanaka K. Lobsiger C. S. Copeland N. G. Jenkins N. A. Kassiotis G.. (2006). Onset and progression in inherited ALS determined by motor neurons and microglia. Science 312, 1389–1392. 10.1126/science.1123511 16741123

[B14] Carson J. H. Barbarese E. (2005). Systems analysis of RNA trafficking in neural cells. Biol. Cell 97, 51–62. 10.1042/BC20040083 15601257

[B15] Chan C. C. Scoggin S. Wang D. Cherry S. Dembo T. Greenberg B.. (2011). Systematic discovery of Rab GTPases with synaptic functions in Drosophila. Curr. Biol. 21, 1704–1715. 10.1016/j.cub.2011.08.058 22000105 PMC3351199

[B16] Chang C. Lang H. Geng N. Wang J. Li N. Wang X. (2013). Exosomes of BV-2 cells induced by alpha-synuclein: important mediator of neurodegeneration in PD. Neurosci. Lett. 548, 190–195. 10.1016/j.neulet.2013.06.009 23792198

[B17] Chen S. Zhang X. Song L. Le W. (2012). Autophagy dysregulation in amyotrophic lateral sclerosis. Brain Pathol. 22, 110–116. 10.1111/j.1750-3639.2011.00546.x 22150926 PMC8029048

[B18] Chen X. Liang H. Zhang J. Zen K. Zhang C. Y. (2012). Horizontal transfer of microRNAs: molecular mechanisms and clinical applications. Protein Cell 3, 28–37. 10.1007/s13238-012-2003-z 22314808 PMC4875218

[B19] Chevallier J. Koop C. Srivastava A. Petrie R. J. Lamarche-Vane N. Presley J. F. (2009). Rab35 regulates neurite outgrowth and cell shape. FEBS Lett. 583, 1096–1101. 10.1016/j.febslet.2009.03.012 19289122

[B20] Chevillet J. R. Kang Q. Ruf I. K. Briggs H. A. Vojtech L. N. Hughes S. M.. (2014). Quantitative and stoichiometric analysis of the microRNA content of exosomes. Proc. Natl. Acad. Sci. U.S.A. 111, 14888–14893. 10.1073/pnas.1408301111 25267620 PMC4205618

[B21] Chiasserini D. Van Weering J. R. Piersma S. R. Pham T. V. Malekzadeh A. Teunissen C. E.. (2014). Proteomic analysis of cerebrospinal fluid extracellular vesicles: a comprehensive dataset. J. Proteomics 106, 191–204. 10.1016/j.jprot.2014.04.028 24769233

[B22] Chivet M. Javalet C. Hemming F. Pernet-Gallay K. Laulagnier K. Fraboulet S.. (2013). Exosomes as a novel way of interneuronal communication. Biochem. Soc. Trans. 41, 241–244. 10.1042/BST20120266 23356290

[B23] Chutna O. Goncalves S. Villar-Pique A. Guerreiro P. Marijanovic Z. Mendes T.. (2014). The small GTPase Rab11 co-localizes with alpha-synuclein in intracellular inclusions and modulates its aggregation, secretion and toxicity. Hum. Mol. Genet. 23, 6732–6745. 10.1093/hmg/ddu391 25092884

[B24] Cocucci E. Meldolesi J. (2015). Ectosomes and exosomes: shedding the confusion between extracellular vesicles. Trends Cell Biol. 25, 364–372. 10.1016/j.tcb.2015.01.004 25683921

[B25] Cocucci E. Racchetti G. Meldolesi J. (2009). Shedding microvesicles: artefacts no more. Trends Cell Biol. 19, 43–51. 10.1016/j.tcb.2008.11.003 19144520

[B26] Coleman B. M. Hill A. F. (2015). Extracellular vesicles–Their role in the packaging and spread of misfolded proteins associated with neurodegenerative diseases. Semin. Cell Dev. Biol. 40, 89–96. 10.1016/j.semcdb.2015.02.007 25704308

[B27] Colombo E. Borgiani B. Verderio C. Furlan R. (2012). Microvesicles: novel biomarkers for neurological disorders. Front. Physiol. 3:63. 10.3389/fphys.2012.00063 22479250 PMC3315111

[B28] Colombo M. Moita C. Van Niel G. Kowal J. Vigneron J. Benaroch P.. (2013). Analysis of ESCRT functions in exosome biogenesis, composition and secretion highlights the heterogeneity of extracellular vesicles. J. Cell Sci. 126, 5553–5565. 10.1242/jcs.128868 24105262

[B29] Colombo M. Raposo G. Théry C. (2014). Biogenesis, secretion, and intercellular interactions of exosomes and other extracellular vesicles. Annu. Rev. Cell Dev. Biol. 30, 255–289. 10.1146/annurev-cellbio-101512-122326 25288114

[B30] Cooper J. M. Wiklander P. B. Nordin J. Z. Al-Shawi R. Wood M. J. Vithlani M.. (2014). Systemic exosomal siRNA delivery reduced alpha-synuclein aggregates in brains of transgenic mice. Movem. Disord. 29, 1476–1485. 10.1002/mds.25978 25112864 PMC4204174

[B31] Danzer K. M. Kranich L. R. Ruf W. P. Cagsal-Getkin O. Winslow A. R. Zhu L.. (2012). Exosomal cell-to-cell transmission of alpha synuclein oligomers. Mol. Neurodegener. 7:42. 10.1186/1750-1326-7-42 22920859 PMC3483256

[B32] Ding X. Ma M. Teng J. Teng R. K. Zhou S. Yin J.. (2015). Exposure to ALS-FTD-CSF generates TDP-43 aggregates in glioblastoma cells through exosomes and TNTs-like structure. Oncotarget 6, 24178–24191. 10.18632/oncotarget.4680 26172304 PMC4695178

[B33] Dinkins M. B. Dasgupta S. Wang G. Zhu G. Bieberich E. (2014). Exosome reduction *in vivo* is associated with lower amyloid plaque load in the 5XFAD mouse model of Alzheimer's disease. Neurobiol. Aging 35, 1792–1800. 10.1016/j.neurobiolaging.2014.02.012 24650793 PMC4035236

[B34] El-Andaloussi S. Lee Y. Lakhal-Littleton S. Li J. Seow Y. Gardiner C.. (2012). Exosome-mediated delivery of siRNA *in vitro* and *in vivo*. Nat. Protoc. 7, 2112–2126. 10.1038/nprot.2012.131 23154783

[B35] Emmanouilidou E. Melachroinou K. Roumeliotis T. Garbis S. D. Ntzouni M. Margaritis L. H.. (2010). Cell-produced alpha-synuclein is secreted in a calcium-dependent manner by exosomes and impacts neuronal survival. J. Neurosci. 30, 6838–6851. 10.1523/JNEUROSCI.5699-09.2010 20484626 PMC3842464

[B36] Etoh K. Fukuda M. (2015). Structure-function analyses of the small GTPase Rab35 and its effector protein centaurin-beta2/ACAP2 during neurite outgrowth of PC12 cells. J. Biol. Chem. 290, 9064–9074. 10.1074/jbc.M114.611301 25694427 PMC4423693

[B37] Fang Y. Wu N. Gan X. Yan W. Morrell J. C. Gould S. J. (2007). Higher-order oligomerization targets plasma membrane proteins and HIV gag to exosomes. PLoS Biol. 5:e158. 10.1371/journal.pbio.0050158 17550307 PMC1885833

[B38] Faure J. Lachenal G. Court M. Hirrlinger J. Chatellard-Causse C. Blot B.. (2006). Exosomes are released by cultured cortical neurones. Mol. Cell. Neurosci. 31, 642–648. 10.1016/j.mcn.2005.12.003 16446100

[B39] Fevrier B. Vilette D. Archer F. Loew D. Faigle W. Vidal M.. (2004). Cells release prions in association with exosomes. Proc. Natl. Acad. Sci. U.S.A. 101, 9683–9688. 10.1073/pnas.0308413101 15210972 PMC470735

[B40] Fiandaca M. S. Kapogiannis D. Mapstone M. Boxer A. Eitan E. Schwartz J. B.. (2015). Identification of preclinical Alzheimer's disease by a profile of pathogenic proteins in neurally derived blood exosomes: a case-control study. Alzheimer's Dement. 11:600–7.e1. 10.1016/j.jalz.2014.06.008 25130657 PMC4329112

[B41] Frey B. Gaipl U. S. (2011). The immune functions of phosphatidylserine in membranes of dying cells and microvesicles. Semin. Immunopathol. 33, 497–516. 10.1007/s00281-010-0228-6 20941495

[B42] Fröhlich D. Kuo W. P. Frühbeis C. Sun J. J. Zehendner C. M. Luhmann H. J.. (2014). Multifaceted effects of oligodendroglial exosomes on neurons: impact on neuronal firing rate, signal transduction and gene regulation. Philos. Trans. R. Soc. Lond. Ser. B Biol. Sci. 369:20130510. 10.1098/rstb.2013.0510 25135971 PMC4142031

[B43] Fruhbeis C. Frohlich D. Kuo W. P. Amphornrat J. Thilemann S. Saab A. S.. (2013). Neurotransmitter-triggered transfer of exosomes mediates oligodendrocyte-neuron communication. PLoS Biol. 11:e1001604. 10.1371/journal.pbio.1001604 23874151 PMC3706306

[B44] Gabrielli M. Battista N. Riganti L. Prada I. Antonucci F. Cantone L.. (2015). Active endocannabinoids are secreted on extracellular membrane vesicles. EMBO Rep. 16, 213–220. 10.15252/embr.201439668 25568329 PMC4328748

[B45] Gan X. Gould S. J. (2011). Identification of an inhibitory budding signal that blocks the release of HIV particles and exosome/microvesicle proteins. Mol. Biol. Cell 22, 817–830. 10.1091/mbc.E10-07-0625 21248205 PMC3057706

[B46] Garden G. A. La Spada A. R. (2012). Intercellular (mis)communication in neurodegenerative disease. Neuron 73, 886–901. 10.1016/j.neuron.2012.02.017 22405200 PMC3334539

[B47] Ghidoni R. Benussi L. Binetti G. (2008). Exosomes: the Trojan horses of neurodegeneration. Med. Hypotheses 70, 1226–1227. 10.1016/j.mehy.2007.12.003 18226468

[B48] Goetzl E. J. Boxer A. Schwartz J. B. Abner E. L. Petersen R. C. Miller B. L.. (2015). Altered lysosomal proteins in neural-derived plasma exosomes in preclinical Alzheimer disease. Neurology 85, 40–47. 10.1212/WNL.0000000000001702 26062630 PMC4501943

[B49] Goldie B. J. Dun M. D. Lin M. Smith N. D. Verrills N. M. Dayas C. V.. (2014). Activity-associated miRNA are packaged in Map1b-enriched exosomes released from depolarized neurons. Nucleic Acids Res. 42, 9195–9208. 10.1093/nar/gku594 25053844 PMC4132720

[B50] Gomes C. Keller S. Altevogt P. Costa J. (2007). Evidence for secretion of Cu,Zn superoxide dismutase via exosomes from a cell model of amyotrophic lateral sclerosis. Neurosci. Lett. 428, 43–46. 10.1016/j.neulet.2007.09.024 17942226

[B51] Gosselin R. D. Meylan P. Decosterd I. (2013). Extracellular microvesicles from astrocytes contain functional glutamate transporters: regulation by protein kinase C and cell activation. Front. Cell. Neurosci. 7:251. 10.3389/fncel.2013.00251 24368897 PMC3857901

[B52] Grad L. I. Yerbury J. J. Turner B. J. Guest W. C. Pokrishevsky E. O'neill M. A.. (2014). Intercellular propagated misfolding of wild-type Cu/Zn superoxide dismutase occurs via exosome-dependent and -independent mechanisms. Proc. Natl. Acad. Sci. U.S.A. 111, 3620–3625. 10.1073/pnas.1312245111 24550511 PMC3948312

[B53] Gross J. C. Chaudhary V. Bartscherer K. Boutros M. (2012). Active Wnt proteins are secreted on exosomes. Nat. Cell Biol. 14, 1036–1045. 10.1038/ncb2574 22983114

[B54] Gu X. Li C. Wei W. Lo V. Gong S. Li S. H.. (2005). Pathological cell-cell interactions elicited by a neuropathogenic form of mutant Huntingtin contribute to cortical pathogenesis in HD mice. Neuron 46, 433–444. 10.1016/j.neuron.2005.03.025 15882643

[B55] Guduric-Fuchs J. O'connor A. Camp B. O'neill C. L. Medina R. J. Simpson D. A. (2012). Selective extracellular vesicle-mediated export of an overlapping set of microRNAs from multiple cell types. BMC Genomics 13:357. 10.1186/1471-2164-13-357 22849433 PMC3532190

[B56] Harrington M. G. Fonteh A. N. Oborina E. Liao P. Cowan R. P. Mccomb G.. (2009). The morphology and biochemistry of nanostructures provide evidence for synthesis and signaling functions in human cerebrospinal fluid. Cerebrospinal Fluid Res. 6:10. 10.1186/1743-8454-6-10 19735572 PMC2746175

[B57] Ilieva H. Polymenidou M. Cleveland D. W. (2009). Non-cell autonomous toxicity in neurodegenerative disorders: ALS and beyond. J. Cell Biol. 187, 761–772. 10.1083/jcb.200908164 19951898 PMC2806318

[B58] Janas T. Janas M. M. Sapon K. (2015). Mechanisms of RNA loading into exosomes. FEBS Lett. 589, 1391–1398. 10.1016/j.febslet.2015.04.036 25937124

[B59] Janas T. Yarus M. (2006). Specific RNA binding to ordered phospholipid bilayers. Nucleic Acids Res. 34, 2128–2136. 10.1093/nar/gkl220 16641318 PMC1449910

[B60] Jeppesen D. K. Hvam M. L. Primdahl-Bengtson B. Boysen A. T. Whitehead B. Dyrskjot L.. (2014). Comparative analysis of discrete exosome fractions obtained by differential centrifugation. J. Extracell. Vesicl. 3:25011. 10.3402/jev.v3.25011 25396408 PMC4224706

[B61] Johnson J. O. Mandrioli J. Benatar M. Abramzon Y. Van Deerlin V. M. Trojanowski J. Q.. (2010). Exome sequencing reveals VCP mutations as a cause of familial ALS. Neuron 68, 857–864. 10.1016/j.neuron.2010.11.036 21145000 PMC3032425

[B62] Joshi P. Turola E. Ruiz A. Bergami A. Libera D. D. Benussi L.. (2014). Microglia convert aggregated amyloid-beta into neurotoxic forms through the shedding of microvesicles. Cell Death Differ. 21, 582–593. 10.1038/cdd.2013.180 24336048 PMC3950321

[B63] Kalra H. Simpson R. J. Ji H. Aikawa E. Altevogt P. Askenase P.. (2012). Vesiclepedia: a compendium for extracellular vesicles with continuous community annotation. PLoS Biol. 10:e1001450. 10.1371/journal.pbio.1001450 23271954 PMC3525526

[B64] Kanada M. Bachmann M. H. Hardy J. W. Frimannson D. O. Bronsart L. Wang A.. (2015). Differential fates of biomolecules delivered to target cells via extracellular vesicles. Proc. Natl. Acad. Sci. U.S.A. 112, E1433–E1442. 10.1073/pnas.1418401112 25713383 PMC4378439

[B65] Kapuralin K. Curlin M. Mitrecic D. Kosi N. Schwarzer C. Glavan G.. (2015). STAM2, a member of the endosome-associated complex ESCRT-0 is highly expressed in neurons. Mol. Cell. Neurosci. 67, 104–115. 10.1016/j.mcn.2015.06.009 26101075

[B66] Keerthikumar S. Chisanga D. Ariyaratne D. Al Saffar H. Anand S. Zhao K.. (2015a). ExoCarta: a web-based compendium of exosomal cargo. J. Mol. Biol. 428, 688–692. 10.1016/j.jmb.2015.09.019 26434508 PMC4783248

[B67] Keerthikumar S. Gangoda L. Liem M. Fonseka P. Atukorala I. Ozcitti C.. (2015b). Proteogenomic analysis reveals exosomes are more oncogenic than ectosomes. Oncotarget 6, 15375–15396. 10.18632/oncotarget.3801 25944692 PMC4558158

[B68] Kim D. K. Lee J. Kim S. R. Choi D. S. Yoon Y. J. Kim J. H.. (2015). EVpedia: a community web portal for extracellular vesicles research. Bioinformatics 31, 933–939. 10.1093/bioinformatics/btu741 25388151 PMC4375401

[B69] Kim H. J. Kim N. C. Wang Y. D. Scarborough E. A. Moore J. Diaz Z.. (2013). Mutations in prion-like domains in hnRNPA2B1 and hnRNPA1 cause multisystem proteinopathy and ALS. Nature 495, 467–473. 10.1038/nature11922 23455423 PMC3756911

[B70] Kim K. Lee S. G. Kegelman T. P. Su Z. Z. Das S. K. Dash R.. (2011). Role of excitatory amino acid transporter-2 (EAAT2) and glutamate in neurodegeneration: opportunities for developing novel therapeutics. J. Cell. Physiol. 226, 2484–2493. 10.1002/jcp.22609 21792905 PMC3130100

[B71] Kobayashi H. Etoh K. Fukuda M. (2014). Rab35 is translocated from Arf6-positive perinuclear recycling endosomes to neurite tips during neurite outgrowth. Small GTPases 5, e29290. 10.4161/sgtp.29290 24852758 PMC4114588

[B72] Kobayashi H. Fukuda M. (2012). Rab35 regulates Arf6 activity through centaurin-beta2 (ACAP2) during neurite outgrowth. J. Cell Sci. 125, 2235–2243. 10.1242/jcs.098657 22344257

[B73] Kobayashi H. Fukuda M. (2013). Rab35 establishes the EHD1-association site by coordinating two distinct effectors during neurite outgrowth. J. Cell Sci. 126, 2424–2435. 10.1242/jcs.117846 23572513

[B74] Korkut C. Li Y. Koles K. Brewer C. Ashley J. Yoshihara M.. (2013). Regulation of postsynaptic retrograde signaling by presynaptic exosome release. Neuron 77, 1039–1046. 10.1016/j.neuron.2013.01.013 23522040 PMC3626103

[B75] Kramer-Albers E. M. Bretz N. Tenzer S. Winterstein C. Mobius W. Berger H.. (2007). Oligodendrocytes secrete exosomes containing major myelin and stress-protective proteins: Trophic support for axons? Proteomics. Clin. Appl. 1, 1446–1461. 10.1002/prca.200700522 21136642

[B76] Lachenal G. Pernet-Gallay K. Chivet M. Hemming F. J. Belly A. Bodon G.. (2011). Release of exosomes from differentiated neurons and its regulation by synaptic glutamatergic activity. Mol. Cell. Neurosci. 46, 409–418. 10.1016/j.mcn.2010.11.004 21111824

[B77] Lai C. P. Mardini O. Ericsson M. Prabhakar S. Maguire C. A. Chen J. W.. (2014a). Dynamic biodistribution of extracellular vesicles *in vivo* using a multimodal imaging reporter. ACS Nano 8, 483–494. 10.1021/nn404945r 24383518 PMC3934350

[B78] Lai C. P. Tannous B. A. Breakefield X. O. (2014b). Noninvasive *in vivo* monitoring of extracellular vesicles. Methods Mol. Biol. 1098, 249–258. 10.1007/978-1-62703-718-1_19 24166382 PMC5798617

[B79] Lauranzano E. Pozzi S. Pasetto L. Stucchi R. Massignan T. Paolella K.. (2015). Peptidylprolyl isomerase A governs TARDBP function and assembly in heterogeneous nuclear ribonucleoprotein complexes. Brain 138, 974–991. 10.1093/brain/awv005 25678563

[B80] Lotvall J. Hill A. F. Hochberg F. Buzas E. I. Di Vizio D. Gardiner C.. (2014). Minimal experimental requirements for definition of extracellular vesicles and their functions: a position statement from the International Society for Extracellular Vesicles. J. Extracell. Vesicl. 3:26913. 10.3402/jev.v3.26913 25536934 PMC4275645

[B81] Lugli G. Cohen A. M. Bennett D. A. Shah R. C. Fields C. J. Hernandez A. G.. (2015). Plasma exosomal miRNAs in persons with and without Alzheimer Disease: altered expression and prospects for biomarkers. PLoS ONE 10:e0139233. 10.1371/journal.pone.0139233 26426747 PMC4591334

[B82] Maas S. L. De Vrij J. Van Der Vlist E. J. Geragousian B. Van Bloois L. Mastrobattista E.. (2015). Possibilities and limitations of current technologies for quantification of biological extracellular vesicles and synthetic mimics. J. Contr. Release. 200, 87–96. 10.1016/j.jconrel.2014.12.041 25555362 PMC4324667

[B83] Marimpietri D. Petretto A. Raffaghello L. Pezzolo A. Gagliani C. Tacchetti C.. (2013). Proteome profiling of neuroblastoma-derived exosomes reveal the expression of proteins potentially involved in tumor progression. PLoS ONE 8:e75054. 10.1371/journal.pone.0075054 24069378 PMC3777909

[B84] Mathivanan S. Simpson R. J. (2009). ExoCarta: a compendium of exosomal proteins and RNA. Proteomics 9, 4997–5000. 10.1002/pmic.200900351 19810033

[B85] Meissner L. Gallozzi M. Balbi M. Schwarzmaier S. M. Tiedt S. Terpolilli N. A. (2015). Temporal profile of microRNA expression in contused cortex following traumatic brain injury in mice. J. Neurotrauma. [Epub ahead of print]. 10.1089/neu.2015.4077 26426744

[B86] Morel L. Regan M. Higashimori H. Ng S. K. Esau C. Vidensky S.. (2013). Neuronal exosomal miRNA-dependent translational regulation of astroglial glutamate transporter GLT1. J. Biol. Chem. 288, 7105–7116. 10.1074/jbc.M112.410944 23364798 PMC3591620

[B87] Muralidharan-Chari V. Clancy J. Plou C. Romao M. Chavrier P. Raposo G.. (2009). ARF6-regulated shedding of tumor cell-derived plasma membrane microvesicles. Curr. Biol. 19, 1875–1885. 10.1016/j.cub.2009.09.059 19896381 PMC3150487

[B88] Nardo G. Pozzi S. Pignataro M. Lauranzano E. Spano G. Garbelli S.. (2011). Amyotrophic lateral sclerosis multiprotein biomarkers in peripheral blood mononuclear cells. PLoS ONE 6:e25545. 10.1371/journal.pone.0025545 21998667 PMC3187793

[B89] Nolte-'T Hoen E. N. Van Der Vlist E. J. Aalberts M. Mertens H. C. Bosch B. J. Bartelink W.. (2012). Quantitative and qualitative flow cytometric analysis of nanosized cell-derived membrane vesicles. Nanomedicine 8, 712–720. 10.1016/j.nano.2011.09.006 22024193 PMC7106164

[B90] Nonaka T. Masuda-Suzukake M. Arai T. Hasegawa Y. Akatsu H. Obi T.. (2013). Prion-like properties of pathological TDP-43 aggregates from diseased brains. Cell Rep. 4, 124–134. 10.1016/j.celrep.2013.06.007 23831027

[B91] Ostrowski M. Carmo N. B. Krumeich S. Fanget I. Raposo G. Savina A.. (2010). Rab27a and Rab27b control different steps of the exosome secretion pathway. Nat. Cell Biol. 12, 19–30. 10.1038/ncb2000 19966785

[B92] Potolicchio I. Carven G. J. Xu X. Stipp C. Riese R. J. Stern L. J.. (2005). Proteomic analysis of microglia-derived exosomes: metabolic role of the aminopeptidase CD13 in neuropeptide catabolism. J. Immunol. 175, 2237–2243. 10.4049/jimmunol.175.4.2237 16081791

[B93] Proia P. Schiera G. Mineo M. Ingrassia A. M. Santoro G. Savettieri G.. (2008). Astrocytes shed extracellular vesicles that contain fibroblast growth factor-2 and vascular endothelial growth factor. Int. J. Mol. Med. 21, 63–67. 10.3892/ijmm.21.1.63 18097617

[B94] Rajendran L. Honsho M. Zahn T. R. Keller P. Geiger K. D. Verkade P.. (2006). Alzheimer's disease beta-amyloid peptides are released in association with exosomes. Proc. Natl. Acad. Sci. U.S.A. 103, 11172–11177. 10.1073/pnas.0603838103 16837572 PMC1544060

[B95] Ramachandran S. Palanisamy V. (2012). Horizontal transfer of RNAs: exosomes as mediators of intercellular communication. Wiley Interdiscipl. Rev. RNA 3, 286–293. 10.1002/wrna.115 22012863 PMC3263325

[B96] Rao P. Benito E. Fischer A. (2013). MicroRNAs as biomarkers for CNS disease. Front. Mol. Neurosci. 6:39. 10.3389/fnmol.2013.00039 24324397 PMC3840814

[B97] Saman S. Kim W. Raya M. Visnick Y. Miro S. Jackson B.. (2012). Exosome-associated tau is secreted in tauopathy models and is selectively phosphorylated in cerebrospinal fluid in early Alzheimer disease. J. Biol. Chem. 287, 3842–3849. 10.1074/jbc.M111.277061 22057275 PMC3281682

[B98] Savina A. Fader C. M. Damiani M. T. Colombo M. I. (2005). Rab11 promotes docking and fusion of multivesicular bodies in a calcium-dependent manner. Traffic 6, 131–143. 10.1111/j.1600-0854.2004.00257.x 15634213

[B99] Savina A. Vidal M. Colombo M. I. (2002). The exosome pathway in K562 cells is regulated by Rab11. J. Cell Sci. 115, 2505–2515. 12045221 10.1242/jcs.115.12.2505

[B100] Sbai O. Ould-Yahoui A. Ferhat L. Gueye Y. Bernard A. Charrat E.. (2010). Differential vesicular distribution and trafficking of MMP-2, MMP-9, and their inhibitors in astrocytes. Glia 58, 344–366. 10.1002/glia.20927 19780201

[B101] Schiera G. Proia P. Alberti C. Mineo M. Savettieri G. Di Liegro I. (2007). Neurons produce FGF2 and VEGF and secrete them at least in part by shedding extracellular vesicles. J. Cell. Mol. Med. 11, 1384–1394. 10.1111/j.1582-4934.2007.00100.x 18205708 PMC4401300

[B102] Shi M. Liu C. Cook T. J. Bullock K. M. Zhao Y. Ginghina C.. (2014). Plasma exosomal alpha-synuclein is likely CNS-derived and increased in Parkinson's disease. Acta Neuropathol. 128, 639–650. 10.1007/s00401-014-1314-y 24997849 PMC4201967

[B103] Soo C. Y. Song Y. Zheng Y. Campbell E. C. Riches A. C. Gunn-Moore F.. (2012). Nanoparticle tracking analysis monitors microvesicle and exosome secretion from immune cells. Immunology 136, 192–197. 10.1111/j.1365-2567.2012.03569.x 22348503 PMC3403268

[B104] Stenmark H. (2009). Rab GTPases as coordinators of vesicle traffic. Nat. Rev. Mol. Cell Biol. 10, 513–525. 10.1038/nrm2728 19603039

[B105] Street J. M. Barran P. E. Mackay C. L. Weidt S. Balmforth C. Walsh T. S.. (2012). Identification and proteomic profiling of exosomes in human cerebrospinal fluid. J. Transl. Med. 10:5. 10.1186/1479-5876-10-5 22221959 PMC3275480

[B106] Tomlinson P. R. Zheng Y. Fischer R. Heidasch R. Gardiner C. Evetts S.. (2015). Identification of distinct circulating exosomes in Parkinson's disease. Ann. Clin. Trans. Neurol. 2, 353–361. 10.1002/acn3.175 25909081 PMC4402081

[B107] Trajkovic K. Hsu C. Chiantia S. Rajendran L. Wenzel D. Wieland F.. (2008). Ceramide triggers budding of exosome vesicles into multivesicular endosomes. Science 319, 1244–1247. 10.1126/science.1153124 18309083

[B108] Turola E. Furlan R. Bianco F. Matteoli M. Verderio C. (2012). Microglial microvesicle secretion and intercellular signaling. Front. Physiol. 3:149. 10.3389/fphys.2012.00149 22661954 PMC3357554

[B109] Valadi H. Ekstrom K. Bossios A. Sjostrand M. Lee J. J. Lotvall J. O. (2007). Exosome-mediated transfer of mRNAs and microRNAs is a novel mechanism of genetic exchange between cells. Nat. Cell Biol. 9, 654–659. 10.1038/ncb1596 17486113

[B110] Vella L. J. Greenwood D. L. Cappai R. Scheerlinck J. P. Hill A. F. (2008a). Enrichment of prion protein in exosomes derived from ovine cerebral spinal fluid. Vet. Immunol. Immunopathol. 124, 385–393. 10.1016/j.vetimm.2008.04.002 18501435

[B111] Vella L. J. Sharples R. A. Lawson V. A. Masters C. L. Cappai R. Hill A. F. (2007). Packaging of prions into exosomes is associated with a novel pathway of PrP processing. J. Pathol. 211, 582–590. 10.1002/path.2145 17334982

[B112] Vella L. J. Sharples R. A. Nisbet R. M. Cappai R. Hill A. F. (2008b). The role of exosomes in the processing of proteins associated with neurodegenerative diseases. Eur. Biophys. J. 37, 323–332. 10.1007/s00249-007-0246-z 18064447

[B113] Verderio C. Muzio L. Turola E. Bergami A. Novellino L. Ruffini F.. (2012). Myeloid microvesicles are a marker and therapeutic target for neuroinflammation. Ann. Neurol. 72, 610–624. 10.1002/ana.23627 23109155

[B114] Vidal M. Mangeat P. Hoekstra D. (1997). Aggregation reroutes molecules from a recycling to a vesicle-mediated secretion pathway during reticulocyte maturation. J. Cell Sci. 110 (Pt 16), 1867–1877. 9296387 10.1242/jcs.110.16.1867

[B115] Villarroya-Beltri C. Baixauli F. Gutierrez-Vazquez C. Sanchez-Madrid F. Mittelbrunn M. (2014). Sorting it out: regulation of exosome loading. Semin. Cancer Biol. 28, 3–13. 10.1016/j.semcancer.2014.04.009 24769058 PMC4640178

[B116] Villarroya-Beltri C. Gutierrez-Vazquez C. Sanchez-Cabo F. Perez-Hernandez D. Vazquez J. Martin-Cofreces N.. (2013a). Sumoylated hnRNPA2B1 controls the sorting of miRNAs into exosomes through binding to specific motifs. Nat. Commun. 4, 2980. 10.1038/ncomms3980 24356509 PMC3905700

[B117] Villarroya-Beltri C. Gutierrez-Vazquez C. Sanchez-Cabo F. Perez-Hernandez D. Vazquez J. Martin-Cofreces N.. (2013b). Sumoylated hnRNPA2B1 controls the sorting of miRNAs into exosomes through binding to specific motifs. Nat. Commun. 4, 2980. 10.1038/ncomms3980 24356509 PMC3905700

[B118] Wang G. Dinkins M. He Q. Zhu G. Poirier C. Campbell A.. (2012). Astrocytes secrete exosomes enriched with proapoptotic ceramide and prostate apoptosis response 4 (PAR-4): potential mechanism of apoptosis induction in Alzheimer disease (AD). J. Biol. Chem. 287, 21384–21395. 10.1074/jbc.M112.340513 22532571 PMC3375560

[B119] Wang S. Cesca F. Loers G. Schweizer M. Buck F. Benfenati F.. (2011). Synapsin I is an oligomannose-carrying glycoprotein, acts as an oligomannose-binding lectin, and promotes neurite outgrowth and neuronal survival when released via glia-derived exosomes. J. Neurosci. 31, 7275–7290. 10.1523/JNEUROSCI.6476-10.2011 21593312 PMC6622588

[B120] Wiklander O. P. Nordin J. Z. O'loughlin A. Gustafsson Y. Corso G. Mager I.. (2015). Extracellular vesicle *in vivo* biodistribution is determined by cell source, route of administration and targeting. J. Extracell. Vesicl. 4:26316. 10.3402/jev.v4.26316 25899407 PMC4405624

[B121] Yamanaka K. Boillee S. Roberts E. A. Garcia M. L. Mcalonis-Downes M. Mikse O. R.. (2008). Mutant SOD1 in cell types other than motor neurons and oligodendrocytes accelerates onset of disease in ALS mice. Proc. Natl. Acad. Sci. U.S.A. 105, 7594–7599. 10.1073/pnas.0802556105 18492803 PMC2396671

[B122] Yang Y. Keene C. D. Peskind E. R. Galasko D. R. Hu S. C. Cudaback E.. (2015). Cerebrospinal Fluid Particles in Alzheimer Disease and Parkinson Disease. J. Neuropathol. Exp. Neurol. 74, 672–687. 10.1097/NEN.0000000000000207 26083568 PMC4471879

[B123] Yuyama K. Sun H. Sakai S. Mitsutake S. Okada M. Tahara H.. (2014). Decreased amyloid-beta pathologies by intracerebral loading of glycosphingolipid-enriched exosomes in Alzheimer model mice. J. Biol. Chem. 289, 24488–24498. 10.1074/jbc.M114.577213 25037226 PMC4148874

[B124] Yuyama K. Sun H. Usuki S. Sakai S. Hanamatsu H. Mioka T.. (2015). A potential function for neuronal exosomes: sequestering intracerebral amyloid-beta peptide. FEBS Lett. 589, 84–88. 10.1016/j.febslet.2014.11.027 25436414

